# AN INTERPROFESSIONAL APPROACH TO DEPRESCRIBING: A CURRICULAR FRAMEWORK

**DOI:** 10.1093/geroni/igac059.2027

**Published:** 2022-12-20

**Authors:** Winnie Sun, Cheryl Sadowski, Barb Farrell

**Affiliations:** Ontario Tech University, Lindsay, Ontario, Canada; University of Alberta, Edmonton, Alberta, Canada; Bruyere Continuing Care, Ottawa, Ontario, Canada

## Abstract

Deprescribing is an important approach for managing polypharmacy and reducing harm from potentially inappropriate medications. Healthcare professionals identify barriers to deprescribing, including lack of knowledge and skill. This is not surprising as pre-licensure education does not consistently incorporate components of deprescribing into curricula. As such, there is a clear need to consider how to promote deprescribing competencies, teach related knowledge and skills and assess learning outcomes. The Canadian Deprescribing Network (CaDeN) Health Care Professional Committee undertook a consensus process to develop a proposed competency framework that describes essential knowledge, teaching strategies, and assessment protocols to promote deprescribing skills and advocate for consistent education about deprescribing principles and practices. The framework is informed by the deprescribing process, which includes gathering and interpreting patients’ medication history and clinical information within their context, using tools that help identify potentially inappropriate medications, weighing potential benefit and harm of continuing or deprescribing medications, using shared decision-making to make decisions about deprescribing, communicating deprescribing and monitoring plans, and monitoring progress and outcomes. The competency framework considers interprofessional learning and how to involve patients and care partners in deprescribing decisions. Integrating deprescribing competencies in healthcare curricula requires an intentional and structured approach across all years of the program, focusing on interprofessional collaboration. Learning activities should be active and practical, progressing from early to advanced learner skills and include integration of deprescribing during experiential education. This framework includes a review of the competencies, learning outcomes, and assessment strategies, with a discussion of strategies to incorporate interprofessional learning activities.

